# LDL‐C and hs‐CRP Jointly Modify the Effect of Lp(a) on 5‐Year Death in Patients With Percutaneous Coronary Intervention

**DOI:** 10.1002/clc.70025

**Published:** 2024-10-21

**Authors:** Jiawen Li, Kailun Yan, Pei Zhu, Xiaofang Tang, Yuejin Yang, Runlin Gao, Jinqing Yuan, Xueyan Zhao

**Affiliations:** ^1^ State Key Laboratory of Cardiovascular Disease, Department of Cardiology, National Clinical Research Center for Cardiovascular Diseases, Fu Wai Hospital, National Center for Cardiovascular Diseases Chinese Academy of Medical Sciences and Peking Union Medical College Beijing China

**Keywords:** high‐sensitivity C‐reactive protein, lipoprotein (a), low‐density lipoprotein cholesterol

## Abstract

**Background:**

Recent studies have suggested that adverse events associated with lipoprotein(a) [Lp(a)] might be modified by low‐density lipoprotein cholesterol (LDL‐C) or high‐sensitivity C‐reactive protein (hs‐CRP) levels, but whether LDL‐C and hs‐CRP jointly mediate the outcome of Lp(a) remains unknown in patients with coronary artery disease.

**Methods and Results:**

A prospective study was conducted, enrolling consecutive 10 724 patients with percutaneous coronary intervention (PCI) in 2013. The endpoint event was all‐cause death. A total of 10 000 patients with complete baseline data were finally included. During a median follow‐up of 5.1 years, Lp(a) ≥ 30 mg/dL was an independent risk factor of all‐cause death in the overall population, LDL‐C ≥ 70 mg/dL, and hs‐CRP ≥ 2 mg/L population, respectively. According to concurrent LDL‐C (70 mg/dL) and hs‐CRP (2 mg/L) levels, further analysis revealed that when LDL‐C < 70 mg/dL regardless of hs‐CRP levels, Lp(a) ≥ 30 mg/dL was not an independent predictor of all‐cause death. However, when LDL‐C ≥ 70 mg/dL, Lp(a) ≥ 30 mg/dL was independently associated with a higher risk of all‐cause death in hs‐CRP ≥ 2 mg/L (HR: 1.488, 95% CI: 1.059‒2.092), but not in hs‐CRP < 2 mg/L (HR: 1.303, 95% CI: 0.914‒1.856).

**Conclusion:**

Among PCI patients, Lp(a)‐associated outcome was jointly affected by LDL‐C and hs‐CRP. As long as LDL‐C is well controlled, the adverse effects of increased Lp(a) on cardiovascular risk seem to be weakened, and only when LDL‐C and hs‐CRP increase at the same time, elevated Lp(a) is associated with poorer long‐term outcome.

## Graphical abstract

This large‐scale observational study with a median follow‐up of 5.1 years demonstrated only when LDL‐C and hs‐CRP increased concurrently, elevated Lp(a) was associated poorer long‐term outcome. Our preliminary and exploratory findings may provide a novel perspective for future precise use of specific Lp(a)‐lowering treatments.

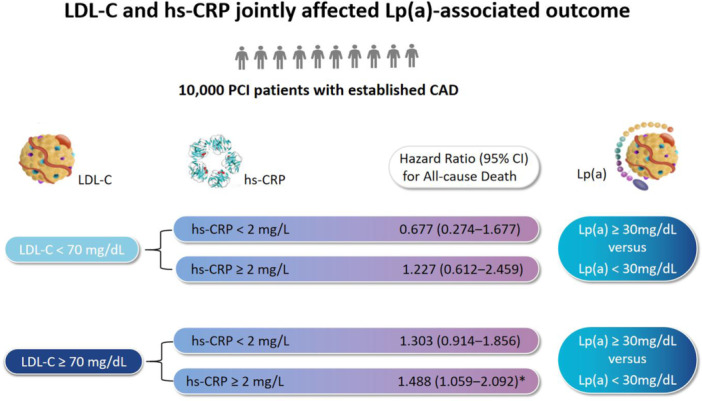

**Abbreviations**
Apo A1: apolipoprotein A1
Apo B: apolipoprotein B
ASCVD: atherosclerotic cardiovascular disease
BMI: body mass index
CAD: coronary artery disease
CI: confidence interval
eGFR: estimated glomerular filtration rate
HDL‐C: high‐density lipoprotein cholesterol
HR: hazard ratio
hs‐CRP: high‐sensitivity C‐reactive protein
IQR: interquartile range
LDL‐C: low‐density lipoprotein cholesterol
Lp(a): lipoprotein(a)
PCI: percutaneous coronary intervention
TC: total cholesterol
TG: triglyceride

## 1 Introduction

Lipoprotein(a) [Lp(a)], as an independent risk factor of adverse events of coronary artery disease (CAD), has been widely concerned in recent years [1]. At present, drugs that specifically reduce Lp(a) are undergoing phase II and III clinical trials with the ability to reduce Lp(a) levels by over 80% (NCT04023552) [2, 3]. Clinically, the accurate identification of the patient population most suitable for Lp(a)‐lowering therapy allows for the supply of more cost‐effective treatment and the achievement of the maximum cardiovascular benefits. However, it is not clear which patients can really benefit from these specific Lp(a)‐lowering therapy.

Previous investigations have demonstrated that the association between Lp(a) and cardiovascular risk may be significantly influenced by low‐density lipoprotein cholesterol (LDL‐C) [4, 5, 6, 7] or high‐sensitivity C‐reactive protein (hs‐CRP) levels [8, 9, 10]. This suggested that LDL‐C and hs‐CRP may aggravate the adverse effects of Lp(a) on cardiovascular system through synergistic effect. It is well known that hs‐CRP, LDL‐C, and Lp(a) are modifiable risk biomarkers that can be used to understand biology, predict risk, and target cardiovascular interventions. We hypothesized that combined concerns of these three modifiable biomarkers could provide greater information on risk compared to Lp(a) alone. However, to our knowledge, there is a dearth of research exploring whether the effect of Lp(a) on adverse prognosis would be simultaneously modified by LDL‐C and hs‐CRP levels in CAD patients. Consequently, we employed a large‐scale and real‐world secondary prevention cohort to examine whether LDL‐C and inflammation levels collectively affect the long‐term prognostic value of Lp(a) in established CAD patients who underwent percutaneous coronary intervention (PCI).

## 2 Materials and Methods

### 2.1 Study Design and Patients

A total of 10 724 patients who underwent PCI at Fu Wai Hospital (Beijing, China) were consecutively included in this single‐center, prospective, observational study conducted from January 1 to December 31, 2013. Eligible patients had undergone PCI and were willing to undergo follow‐up. There were no pre‐specified exclusion criteria. Baseline characteristics data were obtained from electronic medical records. The study protocol and data collection adhered to the principles of the Declaration of Helsinki and were approved by the Review Board of Fu Wai Hospital (Approval Number: 2021‐1501). Written informed consent was obtained from all participants before the procedure.

### 2.2 Laboratory Testing

Patients admitted to the hospital underwent venous blood sampling following a minimum 12‐h fasting period. Lp(a) levels were measured using the immunoturbidimetry method (Lp(a) Assay Kit; SHIMA Laboratories Co., Ltd., Tokyo, Japan), with a normal value of < 30 mg/dL. Use the optional Lp(a) calibrator to measure them as samples, following the procedure outlined in the introduction of the Lp(a) Assay Kit (https://shimalab.co.jp/en/wp-content/uploads/sites/4/2021/05/LASAYLpa_EN.pdf), and create a calibration line. When measuring control serum of known concentration, the measured value should be within ±10% of the known value. When measuring the same sample five times simultaneously, the coefficient of variation of the absorbance values should be 10% or less. Hs‐CRP levels were determined using the immunoturbidimetry method on a specific protein analyzer (Beckmann Assay 360; Beckman Coulter, Brea, California, USA). LDL‐C levels were measured using a selective melt method (LDL‐C Assay Kit, Kyowa Medex Co., Ltd., Tokyo, Japan) with a coefficient of variation of < 5% and total imprecision of < 10%. High‐density lipoprotein cholesterol (HDL‐C) levels were determined using a chemistry modify enzyme method (Determiner L HDL, Kyowa Medex Co., Ltd., Tokyo, Japan) with a coefficient of variation of < 5% and total imprecision of < 10%. Total cholesterol (TC) levels were measured using the CHOD‐PAP method, triglyceride (TG) levels were measured using the GPO‐PAP method, and glucose levels were measured using the glucose oxidase method, all using commercially available test kits (Biosino Bio‐Technology and Science Incorporation, Beijing, China). Apolipoprotein A1 (Apo A1) and apolipoprotein B (Apo B) levels were measured using the immunoturbidimetric method (Tina‐quant Apo A‐1/Apo B ver.2, Roche Diagnostics reagents, Mannheim, Germany). Serum creatinine was determined using the creatinine assay kit with the sarcosine oxidase method (Weihai Weigao Biotech Co., Ltd., Shandong, China). The estimated glomerular filtration rate (eGFR) was calculated using the CKD‐EPI formula. Analyses were performed on an automatic biochemical analyzer, the HITACHI 7150 (Hitachi Ltd., Hitachi, Japan).

### 2.3 Procedures

In the present study, all patients received PCI according to the contemporaneous practice guidelines in China. Those with selective PCI without long‐term aspirin and clopidogrel therapy were given 300 mg of aspirin and 300 mg of clopidogrel orally at least 24 h before the operation. In the process of PCI, all patients received a definite dose of unfractionated heparin (100 U/kg) and a glycoprotein IIb/IIIa inhibitor if deemed necessary by the operator. Patients who underwent PCI for more than 1 h were given 1000 U heparin sodium additionally.

After the operation, all individuals were administrated aspirin 100 mg daily in combination with either clopidogrel 75 mg daily or ticagrelor 90 mg twice daily for a minimum of 12 months. Experienced interventional cardiologists who are not informed of the research plan did PCI. Upon discharge, all patients without contraindications were prescribed moderate‐intensity statins and dual antiplatelet therapy. The prescribed statins included atorvastatin, simvastatin, rosuvastatin, pivastatin, and fluvastatin. The use of other cardiovascular medications, such as β‐blockers, angiotensin‐converting enzyme inhibitors, or angiotensin‐receptor blockers, was prescribed according to individual patient conditions and the contemporaneous guidelines.

### 2.4 Clinical Outcomes

The endpoint event of interest in this study was all‐cause death. The major adverse cardiovascular events (MACE), included all‐cause death, myocardial infarction (MI), and stroke during follow‐up period. All endpoint events were adjudicated centrally by an independent group of cardiologists. Investigator training, blinded questionnaire filling, and telephone recording were performed to obtain high‐quality data. The diagnostic criteria for MI were in accordance with the third global standard definition. Stroke included ischemic stroke and hemorrhagic stroke. Follow‐up assessments were conducted through clinic visits or telephone interviews. Each patient had at minimum of five follow‐up visits at specific time points: 1 month, 6 months, 1 year, 2 years, and 5 years after PCI.

### 2.5 Statistical Analysis

The threshold of Lp(a) was set at 30 mg/dL, as proposed by the Chinese guidelines for lipid management [11] thereby dividing the subjects into groups with Lp(a) levels < 30 and ≥ 30 mg/dL. The threshold of LDL‐C was set at 70 mg/dL. The threshold of hs‐CRP was set at 2 mg/L in reference to the Canakinumab Anti‐inflammatory Thrombosis Outcome Study (CANTOS) [12].

We used SPSS 23.0 and R Programming Language 4.0.3. If the continuous variable follows a Gaussian distribution, expressed as mean ± standard deviation, if the continuous variable does not follow the Gaussian distribution, expressed as median [interquartile range, (IQR)]. The categorical variables were expressed as frequency (percentage). The continuous data were compared by Student's *t*‐test or Mann–Whitney *U* test; the categorical data were compared by *χ* ^2^ test or Fisher's exact test. The cumulative incidences of clinical outcome (all‐cause death) within 5‐year follow‐up were estimated using Kaplan–Meier approach with log‐rank test. Multivariate Cox proportional‐hazard regression analyses were performed to evaluate the associations between Lp(a) (≥ 30 mg/dL relative to < 30 mg/dL) and long‐term clinical outcome according to each category (LDL‐C and hs‐CRP levels) and their combinations [LDL‐C levels (≥ 70 < 70 mg/dL) and further hs‐CRP levels (≥ 2 mg/L or < 2 mg/L)]. Interactions between LDL‐C, hs‐CRP, and Lp(a) were tested to interpret potential differences. Based on the clinical relevance and their associations with the primary outcome, the covariates getting involved in multivariable‐adjusted model in an all‐enter way were: age, sex, body mass index (BMI), acute coronary syndrome, hypertension, diabetes mellitus, peripheral artery disease, chronic obstructive pulmonary disease, prior MI, prior stroke, prior PCI, prior coronary artery bypass graft, left ventricular ejection fraction, HDL‐C, TG, apo B, glucose, and eGFR. The hazard ratio (HR) and 95% confidence interval (CI) were reported. All statistical analyses were performed at a significance level of two‐sided 0.05.

## 3 Results

### 3.1 Baseline Characteristics

**Figure 1:**
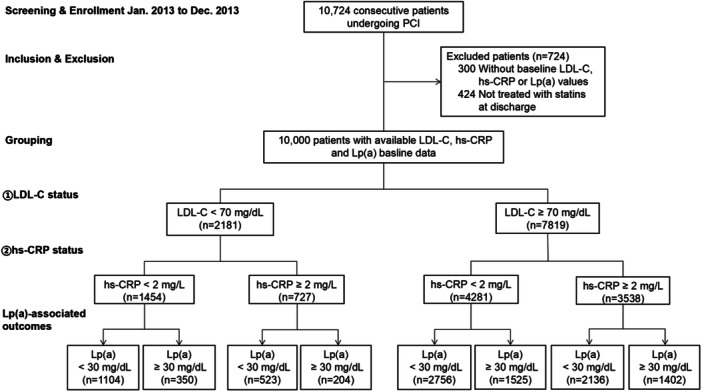
The flow chart of the study. hs‐CRP, high‐sensitivity C‐reactive protein; LDL‐C, low‐density lipoprotein cholesterol; Lp(a), lipoprotein (a); PCI, percutaneous coronary intervention.

A total of consecutive 10 724 CAD patients with PCI were enrolled. After excluding patients without baseline data of LDL‐C, hs‐CRP and Lp(a) and those who did not receive statins treatment, 10 000 (average age 58.33 years, 77.27% male) patients were finally included for analysis (Figure 1). For all participants, the median (IQR) of follow‐up duration was 5.1 (5.0–5.1) years and 358 (3.6%) patients occurred all‐cause death.

The distribution of Lp(a) (18.54 mg/dL, IQR: 7.83‒41.34 mg/dL) differed across LDL‐C categories and hs‐CRP categories (Supporting Information S1: Figure S1). When LDL‐C < 70 mg/dL, the prevalence of elevated Lp(a) [Lp(a) ≥ 30 mg/dL] were 24.1% (350/1454) and 28.1% (204/727), respectively, in patients with hs‐CRP < 2 mg/L and hs‐CRP ≥ 2 mg/L. When LDL‐C ≥ 70 mg/dL, 1525/4281 (35.6%) patients with hs‐CRP < 2 mg/L and 1402/3538 (39.6%) patients with hs‐CRP ≥ 2 mg/L were at Lp(a) ≥ 30 mg/dL, respectively.

The baseline characteristics of the patients stratified by the occurrence of endpoint event are shown in Table 1. Compared with those who survived, the patients with all‐cause death were older, had a lower BMI, had higher rates of hypertension, diabetes mellitus, chronic obstructive pulmonary disease, previous MI, previous stroke, previous PCI, and previous coronary artery bypass graft, had lower levels of left ventricular ejection fraction, TG, and eGFR, and had higher levels of hs‐CRP, HDL‐C, and glucose. The baseline characteristics of patients with Lp(a) levels, LDL‐C levels, and hs‐CRP levels above or below‐specified thresholds are presented in Supporting Information S1: Tables S1–S3, respectively**.** The baseline characteristics in patients with Lp(a) ≥ 30 mg/dL versus Lp(a) < 30 mg/dL across LDL‐C and hs‐CRP categories are shown in Table S4.

**Table 1:** Baseline characteristics of patients stratified by the occurrence of endpoint event.

Variables	Total population (*n* = 10 000)	Without all‐cause death (*n* = 9642)	All‐cause death (*n* = 358)	*p‐*value
Age, years	58.33 ± 10.26	58.08 ± 10.17	65.26 ± 10.33	< 0.001
Sex, %	7727 (77.27)	7461 (77.38)	266 (74.30)	0.193
BMI, kg/m^2^	25.94 ± 3.19	25.95 ± 3.19	25.53 ± 3.18	0.014
Hypertension, %	6438 (64.38)	6175 (64.04)	263 (73.46)	< 0.001
Dyslipidemia, %	6711 (67.11)	6478 (67.19)	233 (65.08)	0.439
Diabetes Mellitus, %	3012 (30.12)	2882 (29.89)	130 (36.31)	0.011
PAD, %	275 (2.75)	259 (2.69)	16 (4.47)	0.063
COPD, %	237 (2.37)	213 (2.21)	24 (6.70)	< 0.001
Previous MI, %	1927 (19.27)	1836 (19.04)	91 (25.42)	0.003
Previous Stroke, %	1059 (10.59)	1008 (10.45)	51 (14.25)	0.028
Current/Ever‐Smoker, %	5852 (58.52)	5640 (58.49)	212 (59.22)	0.827
Previous PCI, %	2418 (24.18)	2307 (23.93)	111 (31.01)	0.003
Previous CABG, %	407 (4.07)	380 (3.94)	27 (7.54)	0.001
Clinical Presentation, %				0.288
Acute coronary syndrome	6796 (67.96)	6543 (67.86)	253 (70.67)	
Stable angina pectoris	3204 (32.04)	3099 (32.14)	105 (29.33)	
LVEF, %	62.82 ± 7.25	62.91 ± 7.14	60.40 ± 9.44	< 0.001
Lp(a), mg/dL	18.54 [7.83, 41.34]	18.49 [7.80, 41.06]	20.97 [8.30, 46.88]	0.052
hs‐CRP, mg/L	1.62 [0.80, 3.73]	1.60 [0.79, 3.68]	2.12 [1.09, 5.42]	< 0.001
LDL‐C, mmol/L	2.51 ± 0.91	2.51 ± 0.91	2.43 ± 0.89	0.097
HDL‐C, mmol/L	1.03 ± 0.28	1.03 ± 0.28	1.07 ± 0.31	0.006
TG, mmol/L	1.79 ± 1.09	1.79 ± 1.09	1.67 ± 0.98	0.034
TC, mmol/L	4.20 ± 1.08	4.21 ± 1.08	4.14 ± 1.06	0.287
Apo A1, g/L	1.35 ± 0.25	1.35 ± 0.25	1.36 ± 0.27	0.303
Apo B, g/L	0.84 ± 0.25	0.84 ± 0.25	0.81 ± 0.24	0.052
Glucose, mmol/L	6.17 ± 2.08	6.15 ± 2.05	6.63 ± 2.77	< 0.001
eGFR, mL/min	91.44 ± 15.02	91.75 ± 14.75	83.01 ± 19.16	< 0.001
Lesion vessels	1.41 ± 0.66	1.41 ± 0.66	1.34 ± 0.61	0.066
SYNTAX score^a^	11.70 ± 8.11	11.69 ± 8.08	11.95 ± 8.92	0.555
Number of stents	1.80 ± 1.11	1.81 ± 1.11	1.75 ± 1.07	0.307
Medication at discharge, %				
Aspirin	9895 (98.95)	9544 (98.98)	351 (98.04)	0.148
Clopidogrel	9980 (99.80)	9623 (99.80)	357 (99.72)	1.000
Calcium channel blocker	4894 (48.94)	4700 (48.75)	194 (54.19)	0.049
Beta‐blocker	9047 (90.47)	8728 (90.52)	319 (89.11)	0.422
Statin	10 000 (100.00)	9642 (100.00)	358 (100.00)	1.000

*Note:* To convert cholesterol to mg/dL, divide values by 0.0259.

Abbreviations: Apo A1: apolipoprotein A1; Apo B: apolipoprotein B; BMI, body mass index; CABG, coronary artery bypass grafting; COPD, chronic obstructive pulmonary disease; eGFR, estimated glomerular filtration rate; HDL‐C, high‐density lipoprotein cholesterol; hs‐CRP, high‐sensitivity C‐reactive protein; LDL‐C, low‐density lipoprotein cholesterol; Lp(a), lipoprotein (a); LVEF, left ventricular ejection fraction; MI, myocardial infarction; PAD, peripheral artery disease; PCI, percutaneous coronary intervention; TC, total cholesterol; TG, triglyceride.

^a^ Calculated using an online calculator (http://www.syntaxscore.com) by a dedicated research group blinded to the clinical data.

### 3.2 The Effect of Lp(a) on Clinical Outcome in the Overall Population

**Figure 2:**
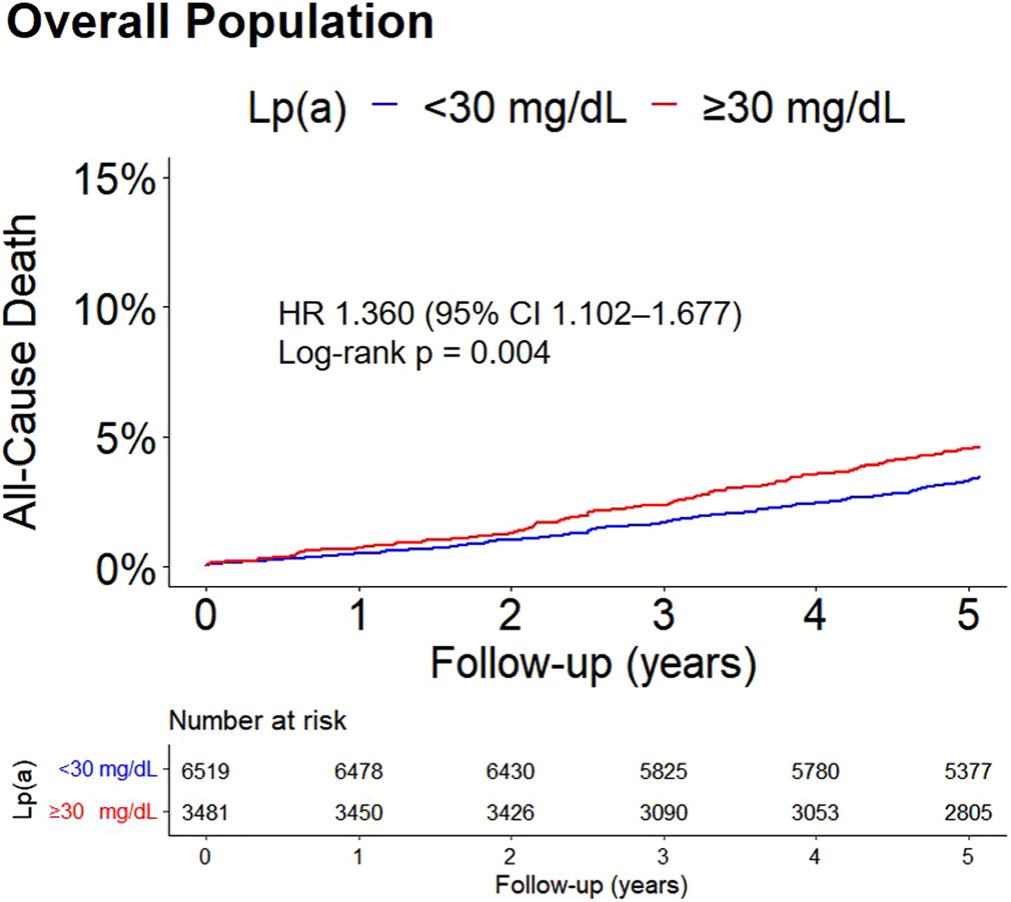
Cumulative incidence rate of all‐cause death with elevated Lp(a) levels in overall population during the 5‐year follow‐up. CI, confidence interval; HR, hazard ratio; Lp(a), lipoprotein (a).

**Figure 3:**
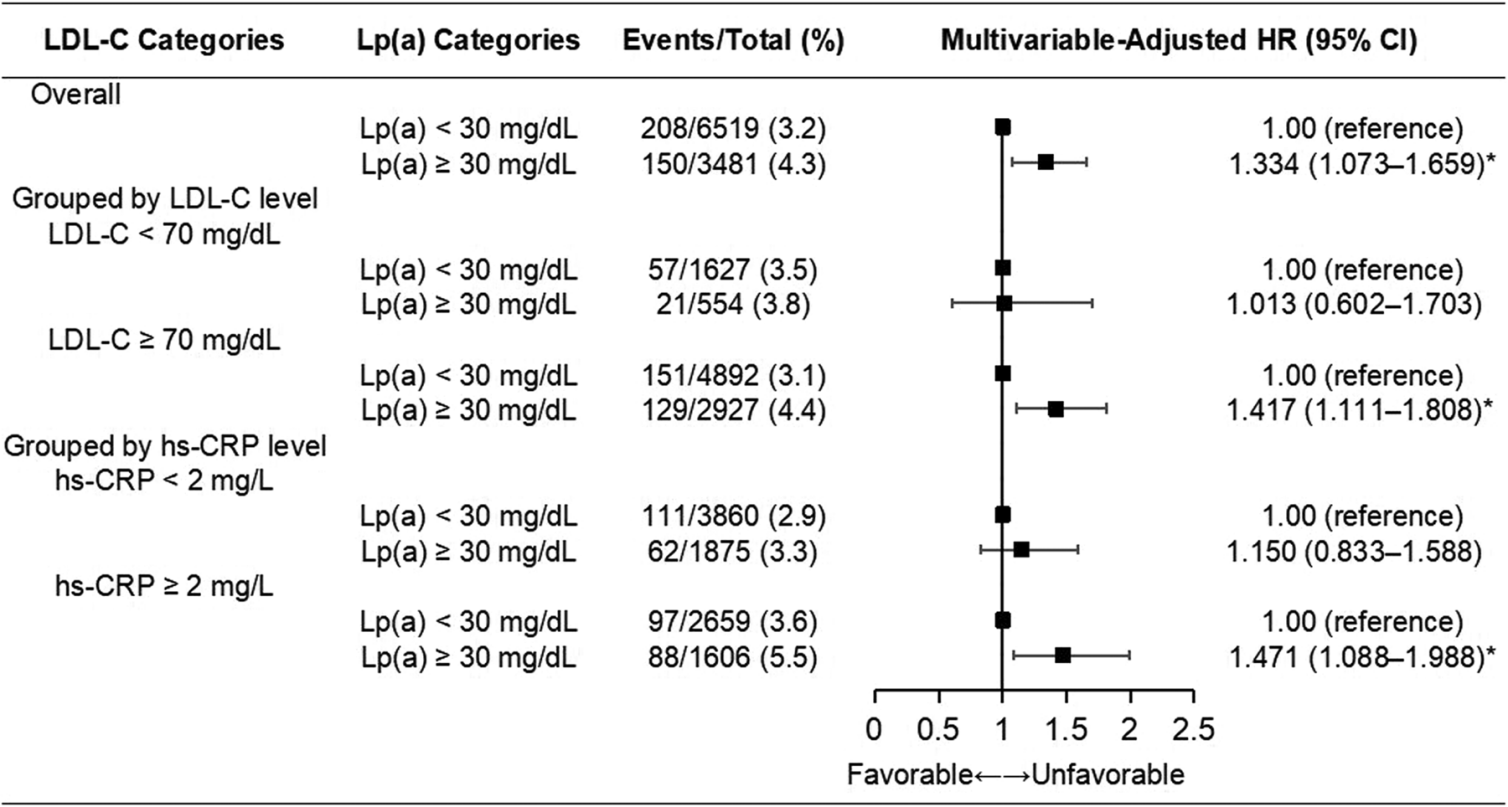
Association of all‐cause death with Lp(a) grouped by LDL‐C or hs‐CRP levels alone. CI, confidence interval; HR, hazard ratio; hs‐CRP, high‐sensitivity C‐reactive protein; LDL‐C, low‐density lipoprotein cholesterol; Lp(a), lipoprotein (a). * *p* < 0.05.

As shown in Figure 2, the Kaplan–Meier analysis shows that patients with Lp(a) ≥ 30 mg/dL had a significantly higher cumulative all‐cause death rate than those with Lp(a) < 30 mg/dL (log‐rank *p* = 0.004). Univariable Cox regression analyses between clinical variables and all‐cause death are shown in Supporting Information S1: Table S5. After multivariable adjustment, the risk of all‐cause death (4.3% vs. 3.2%, adjusted HR: 1.334, 95% CI: 1.073‒1.659) remained significantly higher in Lp(a) ≥ 30 mg/dL relative to Lp(a) < 30 mg/dL in the overall population (Figure 3). The association of Lp(a) and MACE in the overall population is showed in Supporting Information S1: Table S6.

### 3.3 Clinical Outcome Associated With Lp(a) in Relation to LDL‐C or hs‐CRP Alone

Elevated Lp(a) was independently associated with a higher risk of all‐cause death for LDL‐C ≥ 70 mg/dL (4.4% vs. 3.1%, adjusted HR: 1.417, 95% CI: 1.111‒1.808), but not for LDL‐C < 70 mg/dL (3.8% vs. 3.5%, adjusted HR: 1.013, 95% CI: 0.602‒1.703).

Meanwhile, elevated Lp(a) was independently associated with a higher risk of all‐cause death for hs‐CRP ≥ 2 mg/L (5.5% vs. 3.6%, adjusted HR: 1.471, 95% CI: 1.088‒1.988), but not for hs‐CRP < 2 mg/L (3.3% vs. 2.9%, adjusted HR: 1.150, 95% CI: 0.833‒1.588) (Figure 3). The associations of Lp(a) and MACE in relation to LDL‐C or hs‐CRP alone are showed in Supporting Information S1: Table S6.

### 3.4 Lp(a)‐Associated Clinical Outcome According to Combinations (LDL‐C and hs‐CRP Categories)

Notably, there might be a significant interaction for all‐cause death between LDL‐C, hs‐CRP, and Lp(a) (P for interaction = 0.015). The overall population was stratified according to LDL‐C levels (≥ 70 or < 70 mg/dL) and further hs‐CRP levels (≥ 2 mg/L or < 2 mg/L). The Kaplan–Meier curves of the cumulative incidences of all‐cause death with elevated Lp(a) levels according to combinations (LDL‐C and hs‐CRP categories) are shown in Supporting Information S1: Figure S2.

Multivariable Cox regression analyses showed that when LDL‐C < 70 mg/dL, Lp(a) ≥ 30 mg/dL was not an independent risk factor of all‐cause death whether hs‐CRP < 2 mg/L (1.7% vs. 2.8%, adjusted HR: 0.677, 95% CI: 0.274‒1.677) or hs‐CRP ≥ 2 mg/L (7.4% vs. 5.0%, adjusted HR: 1.227, 95% CI: 0.612‒2.459).

**Figure 4:**
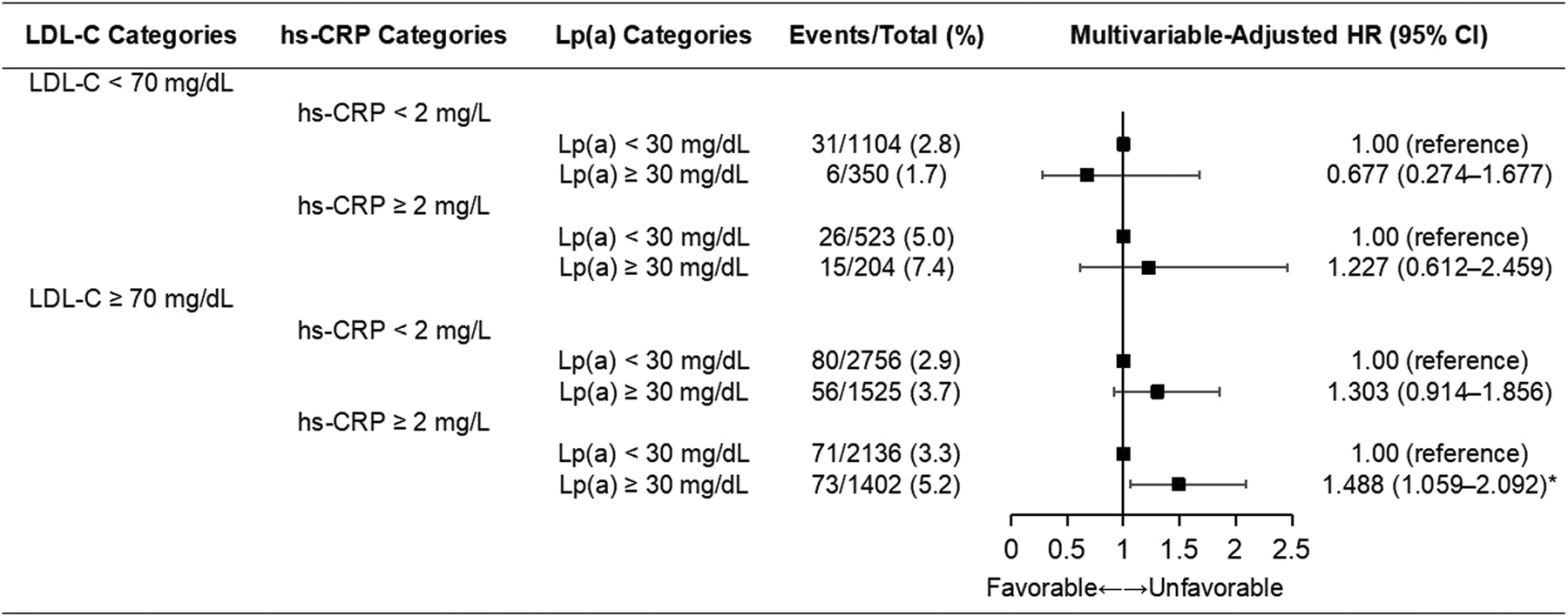
Association of all‐cause death with Lp(a) grouped by concurrent LDL‐C and hs‐CRP levels. CI, confidence interval; HR, hazard ratio; hs‐CRP, high‐sensitivity C‐reactive protein; LDL‐C, low‐density lipoprotein cholesterol; Lp(a), lipoprotein (a); * *p* < 0.05.

Meanwhile, when LDL‐C ≥ 70 mg/dL, Lp(a) ≥ 30 mg/dL remained not an independent risk factor of all‐cause death in the setting of hs‐CRP < 2 mg/L (3.7% vs. 2.9%, adjusted HR: 1.303, 95% CI: 0.914‒1.856). However, when LDL‐C ≥ 70 mg/dL, Lp(a) ≥ 30 mg/dL was an independent risk factor of all‐cause death in the setting of hs‐CRP ≥ 2 mg/L (5.2% vs. 3.3%, adjusted HR: 1.488, 95% CI: 1.059‒2.092) (Figure 4). The associations of Lp(a) and MACE according to LDL‐C and hs‐CRP combination are shown in Supporting Information S1: Table S7.

## 4 Discussion

In this large‐scale observational study with a median follow‐up of 5.1 years, we examined the effect of Lp(a) on long‐term clinical outcome according to different LDL‐C and hs‐CRP levels. The major findings were as follows [1]: Lp(a) ≥ 30 mg/dL was independently associated with the risk of all‐cause death in the overall population, LDL‐C ≥ 70 mg/dL population, and hs‐CRP ≥ 2 mg/L population, respectively [2]; according to the simultaneous levels of LDL‐C and hs‐CRP, further analyses revealed that when LDL‐C < 70 mg/dL, elevated Lp(a) was not an independent predictor of all‐cause death regardless of hs‐CRP levels [3]; only when LDL‐C and hs‐CRP increased concurrently, elevated Lp(a) was associated with poorer long‐term outcome. Our study provides the first report that hs‐CRP and LDL‐C together modified the prognostic value of Lp(a) on long‐term mortality risk in CAD patients. Previous studies have all focused on the individual effect of hs‐CRP or LDL‐C on Lp(a).

### 4.1 The Effect of LDL‐C Levels on Lp(a)‐Associated Outcomes

Previously, a series of research has examined whether Lp(a) remained an independent risk factor for ASCVD or adverse cardiovascular events when LDL‐C concentrations were very low. The GeneBank study (*n* = 2769) has reported a significant attenuation in the correlation between elevated Lp(a) levels (≥ 30 mg/dL) and cardiovascular outcomes (MACE: death, MI, stroke, and coronary revascularization) among patients undergoing coronary angiography when LDL‐C < 70 mg/dL [5]. A large meta‐analysis of 11 secondary prevention trials involving a total of 18,979 individuals has revealed a significant correlation between elevated Lp(a) levels and cardiovascular events (MACE: cardiac death, MI or stroke) in patients with established CAD with an average LDL‐C ≥ 130 mg/dL (OR: 1.46, 95% CI: 1.23–1.73, *p* < 0.001). However, this correlation was not significant in individuals with an average LDL‐C < 130 mg/dL (OR: 1.20, 95% CI: 0.90–1.60, *p* = 0.21) [6]. A small‐scale study (*n* = 516) by Zhu et al. has shown that in patients undergoing PCI due to acute coronary syndrome, elevated Lp(a) levels were significantly associated with an increased risk of cardiovascular events (MACE: cardiac death, nonfatal MI or ischemic stroke, unplanned coronary revascularization, and hospitalization related to unstable angina) when LDL‐C levels were higher (HR: 2.65, 95% CI: 1.31–5.36, *p* = 0.007). Conversely, when LDL‐C levels were extremely low (< 55 mg/dL), no such significant association was observed (HR: 0.49, 95% CI: 0.17–1.42, *p* = 0.186) [7]. In line with the aforementioned research [5, 6, 7], our study supported the notion that the relationship between elevated Lp(a) levels and cardiovascular events may be modified by LDL‐C levels. Of notice, our study focused on those with CAD who received statin (100%) and underwent PCI, and the results indicated that Lp(a) had pathogenicity in those with both high LDL‐C and hs‐CRP level, but appeared to be less atherogenic in patients whose cholesterol level was well controlled (LDL‐C < 70 mg/dL).

### 4.2 The Effect of Hs‐CRP Levels on Lp(a)‐Associated Outcomes

Recent evidence from clinical studies, particularly the CANTOS [12] and Low‐Dose Colchicine (LoDoCo) trials [13] for secondary prevention of CAD, has established the causal role of inflammation in the pathogenesis of ASCAD and related complications. However, limited research has focused on Lp(a)‐associated risk under different hs‐CRP statuses. A prespecified secondary analysis of the Assessment of Clinical Effects of Cholesteryl Ester Transfer Protein Inhibition With Evacetrapib in Patients at a High Risk for Vascular Outcomes (ACCELERATE) trial has demonstrated that Lp(a) held significant prognostic value for cardiovascular event risk (MACE: cardiac death, MI, stroke, coronary revascularization, or hospitalization for unstable angina) in patients with ASCVD only when hs‐CRP levels > 2 mg/L (HR: 1.13, 95% CI: 1.05–1.22, *p* = 0.002) [9]. Similarly, a recent prospective cohort study has found Lp(a) had significant prognostic value for stroke recurrence risk among patients with ischemic stroke or transient ischemic attack only when hs‐CRP > 2 mg/L [10]. Overall, the results in the secondary prevention population have been consistent. Our study yielded analogous findings, indicating that in patients with established CAD, even 100% of them received statins therapy, elevated Lp(a) only had significant prognostic value for cardiovascular event risk in those with hs‐CRP levels > 2 mg/L.

### 4.3 The Joint Effect of LDL‐C and Hs‐CRP Levels on Lp(a)‐Associated Outcomes

There has been a lack of research investigating the predictive value of Lp(a) at different LDL‐C and inflammation statuses together in patients with established CAD undergoing PCI. Our data revealed the significant interdependence among three well‐known cardiovascular risk biomarkers (LDL‐C, hs‐CRP, and Lp[a]). Interestingly, our study demonstrated that when LDL‐C was well‐controlled (on‐treatment LDL‐C < 70 mg/dL), regardless of hs‐CRP levels, the adverse effect of Lp(a) ≥ 30 mg/dL on cardiovascular risk appeared to diminish. This study provided a new perspective into a potential improvement in Lp(a)‐related risk by lowering LDL‐C. However, further randomized clinical studies are still needed to verify whether the pathogenicity of Lp(a) is significantly reduced when the achieved LDL‐C < 70 mg/dL, irrespective of hs‐CRP levels.

The results of our research are explicable, which are consistent with the mainstream recommendations. The Lp(a) consensus statement strongly recommends that Lp(a) should be measured at least once in adults [14]. For patients with elevated Lp(a) levels, there are effective and specific treatments to reduce Lp(a) levels now, and these drugs have successfully progressed to Phase II/III clinical trials. However, it is noteworthy that participants in these trials are limited to patients with atherosclerotic cardiovascular disease or healthy individuals with elevated Lp(a) levels. Given that current research has questioned that Lp(a)‐related outcome may be influenced by LDL‐C and/or hs‐CRP levels, focusing solely on elevated Lp(a) levels may not achieve the maximization of cardiovascular health benefits. It remains unclear which specific patient populations can truly benefit from these Lp(a)‐lowering therapies. Therefore, our research focused on precisely identifying patient whose high risk of cardiovascular events is attributed to elevated Lp(a) levels. Our preliminary findings may provide a novel perspective for developing more cost‐effective treatment strategies, but it is important to note that this research is exploratory, and the conclusions still need to be further validated through prospective, randomized clinical trials. If our study is confirmed, in the future, maybe we can conduct new randomized clinical trials, which will make Lp(a)‐lowering therapies more precise. For example, we can use a specific patient population: CAD patients with both high achieved LDL‐C levels and high inflammatory levels. However, this is only a constructive suggestion.

### 4.4 Potential Mechanisms

The mechanisms underlying the significant interdependence among three cardiovascular risk biomarkers [LDL‐C, hs‐CRP, and Lp[a]] have been not yet fully understood. Our research put forward an exploratory conclusion that only when LDL‐C and hs‐CRP increase simultaneously, the increased Lp(a) is related to poor long‐term prognosis. This suggested that LDL‐C and hs‐CRP may aggravate the adverse effects of Lp(a) on cardiovascular system through synergistic effect. On one hand, Lp(a) is a lipoprotein particle structurally analogous to LDL‐C and has been implicated in promoting atherosclerosis, thrombosis, and inflammation [15]. The effect of excessive Lp(a) on the formation and early development of vascular plaques may accelerate with the presence of other atherogenic lipids such as LDL‐C [16]. Partial involvement of LDL‐receptor in the degradation of Lp(a) has been reported. Increased LDL‐C levels can hinder the breakdown and metabolism of Lp(a) by competing with it for receptor binding, thus intensifying its pathogenic effects [17]. On the other hand, Lp(a) has been identified as a carrier of pro‐inflammatory oxidized phospholipids, indicating its potential pro‐inflammatory properties. This suggested there may be a synergistic effect between elevated Lp(a) and low‐grade inflammation, contributing to the promotion of atherosclerosis [18]. Additionally, the *LPA* gene has a known IL‐6 response element, and hs‐CRP is downstream of IL‐6, indicating that IL‐6 may play a crucial role in the elevation of Lp(a) and hs‐CRP through the inflammatory pathway. It should be noted that the above mechanism is only a hypothetical explanation, and the specific biological mechanism still needs further research to confirm.

### 4.5 Study Limitations

Our study had several limitations. First, it was based on observational and single‐center data which limits the reliability and generalizability of our findings. Second, despite our data being close to a real‐world scenario and our efforts to adjust for important confounding factors, the study may still suffer from residual confounding. Third, in clinical practice, the number of patients with LDL‐C levels controlled below 70 mg/dL following statin treatment in secondary prevention is indeed limited. Although the total population enrolled in our study is relatively large, the low LDL‐C subgroup contains fewer patients due to the real‐world data source, which may face the risk of type II error during hypothesis testing. Meanwhile, different stratification may have different results. A larger sample size is required to further validate our research findings. Fourthly, the duration of statin and the patient's adherence with the lipid‐lowering therapy were not recorded. Lastly, since Lp(a) levels are primarily determined by genetics and vary among different ethnicities, our study was limited to Chinese individuals only, and the results in the other ethnic populations should be further investigated.

## 5 Conclusions

This large‐sample and real‐world study demonstrated that LDL‐C and hs‐CRP jointly mediated the effect of Lp(a) on outcome in patients with CAD after PCI. When LDL‐C was well‐controlled, the adverse effect of elevated Lp(a) on cardiovascular risk appeared to diminish regardless of hs‐CRP levels. The Lp(a)‐associated risk was only in those with concomitant elevation of both LDL‐C and hs‐CRP. Our findings further emphasized in CAD population, LDL‐C and hs‐CRP levels should be focused on together for those with elevated Lp(a). The study may also provide important clues for future precise Lp(a)‐lowering therapy.

## Author Contributions

**Jiawen Li:** conceptualization, methodology, software, data curation, visualization, validation, writing of original draft, funding acquisition. **Kailun Yan:** data curation. **Pei Zhu:** data curation. **Xiaofang Tang:** data curation. **Yuejin Yang:** supervision. **Runlin Gao:** supervision. **Jinqing Yuan:** writing, review, and editing, supervision, funding acquisition. **Xueyan Zhao:** conceptualization, writing, review, and editing, supervision, funding acquisition. All authors agreed to the published version of this manuscript.

## Disclosure

The authors have nothing to report.

## Conflicts of interest

The authors declare no conflicts of interest.

## Supporting information

Supporting information.

## Data Availability

The data that support the findings of this study are available on request from the corresponding author. The data are not publicly available due to privacy or ethical restrictions.

## References

[1] B. G. Nordestgaard and A. Langsted , “Lipoprotein (a) As a Cause of Cardiovascular Disease: Insights From Epidemiology, Genetics, and Biology,” Journal of Lipid Research 57, no. 11 (2016): 1953–1975. 27677946 10.1194/jlr.R071233 PMC5087876

[2] S. Tsimikas , E. Karwatowska‐Prokopczuk , I. Gouni‐Berthold , et al., “Lipoprotein(a) Reduction in Persons With Cardiovascular Disease,” New England Journal of Medicine 382, no. 3 (2020): 244–255. 31893580 10.1056/NEJMoa1905239

[3] M. L. O'Donoghue , R. S. Rosenson , B. Gencer , et al., “Small Interfering RNA to Reduce Lipoprotein(a) in Cardiovascular Disease,” New England Journal of Medicine 387, no. 20 (2022): 1855–1864. 36342163 10.1056/NEJMoa2211023

[4] R. Verbeek , R. M. Hoogeveen , A. Langsted , et al., “Cardiovascular Disease Risk Associated With Elevated Lipoprotein(a) Attenuates at Low Low‐Density Lipoprotein Cholesterol Levels in a Primary Prevention Setting,” European Heart Journal 39, no. 27 (2018): 2589–2596. 29931232 10.1093/eurheartj/ehy334 PMC6287703

[5] S. J. Nicholls , W. H. W. Tang , H. Scoffone , et al., “Lipoprotein(a) Levels and Long‐Term Cardiovascular Risk in the Contemporary Era of Statin Therapy,” Journal of Lipid Research 51, no. 10 (2010): 3055–3061. 20601648 10.1194/jlr.M008961 PMC2936758

[6] M. L. O'Donoghue , D. A. Morrow , S. Tsimikas , et al., “Lipoprotein(a) for Risk Assessment in Patients With Established Coronary Artery Disease,” Journal of the American College of Cardiology 63, no. 6 (2014): 520–527. 24161323 10.1016/j.jacc.2013.09.042 PMC3945105

[7] L. Zhu , J. Zheng , B. Gao , et al., “The Correlation Between Lipoprotein(a) Elevations and the Risk of Recurrent Cardiovascular Events in CAD Patients With Different LDL‐C Levels,” BMC Cardiovascular Disorders 22, no. 1 (2022): 171. 35428179 10.1186/s12872-022-02618-5 PMC9013030

[8] W. Zhang , J. L. Speiser , F. Ye , et al., “High‐Sensitivity C‐Reactive Protein Modifies the Cardiovascular Risk of Lipoprotein(a),” Journal of the American College of Cardiology 78, no. 11 (2021): 1083–1094. 34503676 10.1016/j.jacc.2021.07.016 PMC8444216

[9] R. Puri , S. E. Nissen , B. J. Arsenault , et al., “Effect of C‐Reactive Protein on Lipoprotein(a)‐Associated Cardiovascular Risk in Optimally Treated Patients With High‐Risk Vascular Disease: A Prespecified Secondary Analysis of the ACCELERATE Trial,” JAMA Cardiology 5, no. 10 (2020): 1136–1143. 32639518 10.1001/jamacardio.2020.2413 PMC7344788

[10] J. Xu , X. Hao , R. Zhan , et al., “Effect of Lipoprotein(a) on Stroke Recurrence Attenuates at Low LDL‐C (Low‐Density Lipoprotein) and Inflammation Levels,” Stroke 53, no. 8 (2022): 2504–2511. 35410491 10.1161/STROKEAHA.121.034924

[11] J. Li , S. Zhao , et al., “2023 China Guidelines for Lipid Management,” Journal of Geriatric Cardiology 20, no. 9 (2023): 621–663. 37840633 10.26599/1671-5411.2023.09.008 PMC10568545

[12] P. M. Ridker , B. M. Everett , T. Thuren , et al., “Antiinflammatory Therapy With Canakinumab for Atherosclerotic Disease,” New England Journal of Medicine 377, no. 12 (2017): 1119–1131. 28845751 10.1056/NEJMoa1707914

[13] S. M. Nidorf , J. W. Eikelboom , C. A. Budgeon , and P. L. Thompson , “Low‐Dose Colchicine for Secondary Prevention of Cardiovascular Disease,” Journal of the American College of Cardiology 61, no. 4 (2013): 404–410. 23265346 10.1016/j.jacc.2012.10.027

[14] F. Kronenberg , S. Mora , E. S. G. Stroes , et al., “Lipoprotein(a) in Atherosclerotic Cardiovascular Disease and Aortic Stenosis: A European Atherosclerosis Society Consensus Statement,” European Heart Journal 43, no. 39 (2022): 3925–3946. 36036785 10.1093/eurheartj/ehac361 PMC9639807

[15] S. Tsimikas , “A Test in Context: Lipoprotein(a),” Journal of the American College of Cardiology 69, no. 6 (2017): 692–711. 28183512 10.1016/j.jacc.2016.11.042

[16] M. Afshar , L. Pilote , L. Dufresne , J. C. Engert , and G. Thanassoulis , “Lipoprotein(a) Interactions With Low‐Density Lipoprotein Cholesterol and Other Cardiovascular Risk Factors in Premature Acute Coronary Syndrome (ACS),” Journal of the American Heart Association 5, no. 4 (2016): e003012, 10.1161/JAHA.115.003012. 27108248 PMC4859285

[17] S. L. Hofmann , D. L. Eaton , M. S. Brown , W. J. McConathy , J. L. Goldstein , and R. E. Hammer , “Overexpression of Human Low Density Lipoprotein Receptors Leads to Accelerated Catabolism of Lp(a) Lipoprotein in Transgenic Mice,” Journal of Clinical Investigation 85, no. 5 (1990): 1542–1547. 2139667 10.1172/JCI114602 PMC296603

[18] S. Tsimikas , E. S. Brilakis , E. R. Miller , et al., “Oxidized Phospholipids, Lp(a) Lipoprotein, and Coronary Artery Disease,” New England Journal of Medicine 353, no. 1 (2005): 46–57. 16000355 10.1056/NEJMoa043175

