# Supplementary material for: LDL‐C and hs‐CRP Jointly Modify the Effect of Lp(a) on 5‐Year Death in Patients With Percutaneous Coronary Intervention
Source: Clin Cardiol. 2024 Oct 21;47(10):e70025. doi: 10.1002/clc.70025 (PMC11491544; doi:10.1002/clc.70025)
Supplement: Supplementary file 1 — Supporting information. [file CLC-47-e70025-s001.docx]

**Supplementary Materials**

**LDL-C and hs-CRP jointly modify the effect of Lp(a) on 5-year death in patients with percutaneous coronary intervention**

Jiawen Li^1^, M.D., Kailun Yan^1^, M.D., Pei Zhu^1^, M.D., Xiaofang Tang^1^, M.D., Yuejin Yang^1^, M.D., Runlin Gao^1^, M.D., Jinqing Yuan^1*^, M.D., and Xueyan Zhao^1**^, M.D.

^1^ Department of Cardiology, National Clinical Research Center for Cardiovascular Diseases, State Key Laboratory of Cardiovascular Disease, Fu Wai Hospital, National Center for Cardiovascular Diseases, Chinese Academy of Medical Sciences and Peking Union Medical College, Beijing 100037, China

****Corresponding author.** Xueyan Zhao. Department of Cardiology, Fu Wai Hospital, National Center for Cardiovascular Diseases, Chinese Academy of Medical Sciences, Beijing 100037, China

***Corresponding author.** Jinqing Yuan. Department of Cardiology, Fu Wai Hospital, National Center for Cardiovascular Diseases, Chinese Academy of Medical Sciences, Beijing 100037, China

**Contents**

**Table S1.** Baseline characteristics of patients with Lp(a) ≥ 30 mg/dL versus Lp(a) < 30 mg/dL

**Table S2.** Baseline characteristics of patients with LDL-C ≥ 70 mg/dL versus LDL-C < 70 mg/dL

**Table S3.** Baseline characteristics of patients with hs-CRP ≥ 2 mg/L versus hs-CRP < 2 mg/L

**Table S4.** Baseline characteristics of patients with Lp(a) ≥ 30 mg/dL versus Lp(a) < 30 mg/dL stratified by LDL-C and hs-CRP categories

**Table S5.** Univariable Cox regression for primary outcome

**Table S6.** Association of MACE with Lp(a) grouped by LDL-C or hs-CRP levels alone

**Table S7.** Association of MACE with Lp(a) grouped by concurrent LDL-C and hs-CRP levels

**Figure S1.** Prevalence of Lp(a) < 30 mg/dL and Lp(a) ≥ 30 mg/dL by concurrent LDL-C and hs-CRP categories

**Figure S2.** Cumulative incidence rate of all-cause death with elevated Lp(a) levels according to combinations (LDL-C and hs-CRP categories) during the 5-year follow up.

**Table S1.** Baseline characteristics of patients with Lp(a) ≥ 30 mg/dL versus Lp(a) < 30 mg/dL

| Variables | Lp(a) < 30 mg/dL  (n = 6519) | Lp(a) ≥ 30 mg/dL  (n = 3481) | P value |
| --- | --- | --- | --- |
| Age, years | 58.11 ± 10.33 | 58.76 ± 10.12 | 0.003 |
| Sex, % | 5158 (79.12) | 2569 (73.80) | <0.001 |
| BMI, kg/m^2^ | 26.03 ± 3.20 | 25.77 ± 3.15 | <0.001 |
| Hypertension, % | 4185 (64.20) | 2253 (64.72) | 0.616 |
| Dyslipidemia, % | 4397 (67.45) | 2314 (66.48) | 0.334 |
| Diabetes Mellitus, % | 1999 (30.66) | 1013 (29.10) | 0.110 |
| PAD, % | 177 (2.72) | 98 (2.82) | 0.820 |
| COPD, % | 142 (2.18) | 95 (2.73) | 0.098 |
| Previous MI, % | 1245 (19.10) | 682 (19.59) | 0.569 |
| Previous Stroke, % | 657 (10.08) | 402 (11.55) | 0.025 |
| Current/Ever-Smoker, % | 3905 (59.90) | 1947 (55.93) | <0.001 |
| Previous PCI, % | 1556 (23.87) | 862 (24.76) | 0.332 |
| Previous CABG, % | 234 (3.59) | 173 (4.97) | 0.001 |
| Clinical Presentation, % |  |  | 0.277 |
| Acute Coronary Syndrome | 4455 (68.34) | 2341 (67.25) |  |
| Stable Angina Pectoris | 2064 (31.66) | 1140 (32.75) |  |
| LVEF, % | 62.96 ± 7.12 | 62.56 ± 7.49 | 0.009 |
| Lp(a), mg/dL | 10.41 [5.21, 17.84] | 54.51 [39.89, 79.38] | <0.001 |
| hs-CRP, mg/L | 1.53 [0.76, 3.46] | 1.81 [0.87, 4.40] | <0.001 |
| LDL-C, mmol/L | 2.42 ± 0.89 | 2.66 ± 0.93 | <0.001 |
| HDL-C, mmol/L | 1.02 ± 0.28 | 1.06 ± 0.28 | <0.001 |
| TG, mmol/L | 1.86 ± 1.21 | 1.65 ± 0.79 | <0.001 |
| TC, mmol/L | 4.12 ± 1.07 | 4.36 ± 1.08 | <0.001 |
| Apo A1, g/L | 1.34 ± 0.24 | 1.35 ± 0.26 | 0.067 |
| Apo B, g/L | 0.81 ± 0.24 | 0.90 ± 0.26 | <0.001 |
| Glucose, mmol/L | 6.20 ± 2.08 | 6.12 ± 2.07 | 0.076 |
| eGFR, ml/min | 91.76 ± 14.90 | 90.82 ± 15.24 | 0.003 |
| Lesion Vessels | 1.40 ± 0.66 | 1.43 ± 0.67 | 0.017 |
| SYNTAX Score^a^ | 11.45 ± 7.97 | 12.16 ± 8.34 | <0.001 |
| Number of Stents | 1.79 ± 1.11 | 1.83 ± 1.10 | 0.093 |
| Medication at Discharge, % |  |  |  |
| Aspirin | 6452 (98.97) | 3443 (98.91) | 0.845 |
| Clopidogrel | 6510 (99.86) | 3470 (99.68) | 0.096 |
| Calcium Channel Blocker | 3184 (48.84) | 1710 (49.12) | 0.804 |
| Beta-Blocker | 5861 (89.91) | 3186 (91.53) | 0.010 |
| Statin | 6519 (100.00) | 3481 (100.00) | 1.000 |

BMI, body mass index; PAD, peripheral artery disease; COPD, chronic obstructive pulmonary disease; MI, myocardial infarction; PCI, percutaneous coronary intervention; CABG, coronary artery bypass grafting; LVEF, left ventricular ejection fraction; Lp(a), lipoprotein (a); hs-CRP, high-sensitivity C-reactive protein; LDL-C, low-density lipoprotein cholesterol; HDL-C, high-density lipoprotein cholesterol; TG, triglyceride; TC, total cholesterol; Apo A1: apolipoprotein A1; Apo B: apolipoprotein B; eGFR, estimated glomerular filtration rate.

To convert cholesterol to mg/dL, divide values by 0.0259.

^a^Calculated using an online calculator (http://www.syntaxscore.com) by a dedicated research group blinded to the clinical data.

**Table S2.** Baseline characteristics of patients with LDL-C ≥ 70 mg/dL versus LDL-C < 70 mg/dL

| Variables | LDL-C ≥ 70 mg/dL  (n = 7819) | LDL-C < 70 mg/dL  (n = 2181) | P value |
| --- | --- | --- | --- |
| Age, years | 58.29 ± 10.23 | 58.50 ± 10.37 | 0.394 |
| Sex, % | 5943 (76.01) | 1784 (81.80) | <0.001 |
| BMI, kg/m^2^ | 26.00 ± 3.19 | 25.73 ± 3.18 | 0.001 |
| Hypertension, % | 5007 (64.04) | 1431 (65.61) | 0.182 |
| Dyslipidemia, % | 5256 (67.22) | 1455 (66.71) | 0.674 |
| Diabetes Mellitus, % | 2274 (29.08) | 738 (33.84) | 0.110 |
| PAD, % | 208 (2.66) | 67 (3.07) | 0.334 |
| COPD, % | 184 (2.35) | 53 (2.43) | 0.897 |
| Previous MI, % | 1418 (18.14) | 509 (23.34) | \| <0.001 \| \| --- \| |
| Previous Stroke, % | 802 (10.26) | 257 (11.78) | 0.045 |
| Current/Ever-Smoker, % | 4528 (57.91) | 1324 (60.71) | 0.020 |
| Previous PCI, % | 1787 (22.85) | 631 (28.93) | <0.001 |
| Previous CABG, % | 311 (3.98) | 96 (4.40) | 0.409 |
| Clinical Presentation, % |  |  | 0.824 |
| Acute Coronary Syndrome | 5309 (67.90) | 1487 (68.18) |  |
| Stable Angina Pectoris | 2510 (32.10) | 694 (31.82) |  |
| LVEF, % | 62.77 ± 7.29 | 63.02 ± 7.13 | 0.159 |
| Lp(a), mg/dL | 20.32 [8.65, 44.10] | 12.79 [5.75, 30.33] | <0.001 |
| hs-CRP, mg/L | 1.75 [0.88, 4.04] | 1.20 [0.59, 2.77] | <0.001 |
| LDL-C, mmol/L | 2.79 ± 0.81 | 1.47 ± 0.25 | <0.001 |
| HDL-C, mmol/L | 1.05 ± 0.27 | 0.97 ± 0.28 | <0.001 |
| TG, mmol/L | 1.83 ± 1.01 | 1.63 ± 1.32 | <0.001 |
| TC, mmol/L | 4.52 ± 0.97 | 3.06 ± 0.53 | <0.001 |
| Apo A1, g/L | 1.36 ± 0.25 | 1.28 ± 0.24 | 0.067 |
| Apo B, g/L | 0.91 ± 0.23 | 0.59 ± 0.11 | <0.001 |
| Glucose, mmol/L | 6.24 ± 2.13 | 5.92 ± 1.85 | <0.001 |
| eGFR, ml/min | 91.46 ± 15.05 | 91.34 ± 14.92 | 0.738 |
| Lesion Vessels | 1.42 ± 0.67 | 1.36 ± 0.65 | 0.001 |
| SYNTAX Score^a^ | 11.81 ± 8.14 | 11.27 ± 7.99 | 0.006 |
| Number of Stents | 1.82 ± 1.10 | 1.76 ± 1.12 | 0.037 |
| Medication at Discharge, % |  |  |  |
| Aspirin | 7742 (99.02) | 2153 (98.72) | 0.275 |
| Clopidogrel | 7805 (99.82) | 2175 (99.72) | 0.537 |
| Calcium Channel Blocker | 3841 (49.12) | 1053 (48.28) | 0.501 |
| Beta-Blocker | 7087 (90.64) | 1960 (89.87) | 0.297 |
| Statin | 7819 (100.00) | 2181 (100.00) | 1.000 |

Meanings of the abbreviations are identical to those in Table S1.

^a^Calculated using an online calculator (http://www.syntaxscore.com) by a dedicated research group blinded to the clinical data.

**Table S3.** Baseline characteristics of patients with hs-CRP ≥ 2 mg/L versus hs-CRP < 2 mg/L

| Variables | hs-CRP ≥ 2 mg/L  (n = 4265) | hs-CRP < 2 mg/L  (n = 5735) | P value |
| --- | --- | --- | --- |
| Age, years | 58.60 ± 10.57 | 58.13 ± 10.02 | 0.024 |
| Sex, % | 3240 (75.97) | 4487 (78.24) | 0.008 |
| BMI, kg/m^2^ | 26.31 ± 3.26 | 25.66 ± 3.10 | <0.001 |
| Hypertension, % | 2869 (67.27) | 3569 (62.23) | <0.001 |
| Dyslipidemia, % | 2870 (67.29) | 3841 (66.97) | 0.755 |
| Diabetes Mellitus, % | 1360 (31.89) | 1652 (28.81) | 0.001 |
| PAD, % | 115 (2.70) | 160 (2.79) | 0.825 |
| COPD, % | 132 (3.09) | 105 (1.83) | <0.001 |
| Previous MI, % | 725 (17.00) | 1202 (20.96) | \| <0.001 \| \| --- \| |
| Previous Stroke, % | 505 (11.84) | 554 (9.66) | 0.001 |
| Current/Ever-Smoker, % | 2604 (61.06) | 3248 (56.63) | <0.001 |
| Previous PCI, % | 951 (22.30) | 1467 (25.58) | <0.001 |
| Previous CABG, % | 171 (4.01) | 236 (4.12) | 0.831 |
| Clinical Presentation, % |  |  | <0.001 |
| Acute Coronary Syndrome | 3155 (73.97) | 3641 (63.49) |  |
| Stable Angina Pectoris | 1110 (26.03) | 2094 (36.51) |  |
| LVEF, % | 61.92 ± 7.64 | 63.50 ± 6.88 | <0.001 |
| Lp(a), mg/dL | 21.03 [9.18, 43.63] | 16.63 [6.94, 39.44] | <0.001 |
| hs-CRP, mg/L | 4.49 [2.84, 9.82] | 0.89 [0.53, 1.35] | <0.001 |
| LDL-C, mmol/L | 2.64 ± 0.93 | 2.41 ± 0.89 | <0.001 |
| HDL-C, mmol/L | 0.99 ± 0.26 | 1.07 ± 0.29 | <0.001 |
| TG, mmol/L | 1.88 ± 1.14 | 1.71 ± 1.04 | <0.001 |
| TC, mmol/L | 4.36 ± 1.10 | 4.09 ± 1.05 | <0.001 |
| Apo A1, g/L | 1.31 ± 0.24 | 1.37 ± 0.25 | <0.001 |
| Apo B, g/L | 0.88 ± 0.25 | 0.82 ± 0.24 | <0.001 |
| Glucose, mmol/L | 6.46 ± 2.34 | 5.95 ± 1.83 | <0.001 |
| eGFR, ml/min | 89.96 ± 16.40 | 92.53 ± 13.80 | <0.001 |
| Lesion Vessels | 1.44 ± 0.69 | 1.38 ± 0.64 | <0.001 |
| SYNTAX Score^a^ | 12.23 ± 8.38 | 11.30 ± 7.88 | <0.001 |
| Number of Stents | 1.84 ± 1.14 | 1.78 ± 1.08 | 0.005 |
| Medication at Discharge, % |  |  |  |
| Aspirin | 4217 (98.87) | 5678 (99.01) | 0.590 |
| Clopidogrel | 4257 (99.81) | 5723 (99.79) | 0.989 |
| Calcium Channel Blocker | 2083 (48.84) | 2811 (49.01) | 0.878 |
| Beta-Blocker | 3898 (91.40) | 5149 (89.78) | 0.007 |
| Statin | 3898 (100.00) | 5149 (100.00) | 1.000 |

Meanings of the abbreviations are identical to those in Table S1.

^a^Calculated using an online calculator (http://www.syntaxscore.com) by a dedicated research group blinded to the clinical data.

**Table S4.** Baseline characteristics of patients with Lp(a) ≥ 30 mg/dL versus Lp(a) < 30 mg/dL stratified by LDL-C and hs-CRP categories

| Variables | LDL-C < 70 mg/dL | | | | LDL-C ≥ 70 mg/dL | | | |  |
| --- | --- | --- | --- | --- | --- | --- | --- | --- | --- |
|  | hs-CRP < 2 mg/L | | hs-CRP ≥ 2 mg/L | | hs-CRP < 2 mg/L | | hs-CRP ≥ 2 mg/L | |  |
|  | Lp(a) < 30 mg/dL (n = 1104) | Lp(a) ≥ 30 mg/dL (n= 350) | Lp(a) < 30 mg/dL (n = 523) | Lp(a) ≥ 30 mg/dL (n = 204) | Lp(a) < 30 mg/dL (n = 2756) | Lp(a) ≥ 30 mg/dL (n = 1525) | Lp(a) < 30 mg/dL (n = 2136) | Lp(a) ≥ 30 mg/dL (n = 1402) | P value |
| Age, years | 58.16±10.40 | 58.06±10.05 | 58.63±10.47 | 60.79±10.24 | 57.92±10.12 | 58.52±9.53 | 58.19±10.52 | 58.90±10.70 | 0.002 |
| Sex, % | 916 (82.97) | 285 (81.43) | 424 (81.07) | 159 (77.94) | 2184 (79.25) | 1102 (72.26) | 1634 (76.50) | 1023 (72.97) | <0.001 |
| BMI, kg/m2 | 25.60±3.07 | 25.42±3.10 | 26.20±3.38 | 25.80±3.24 | 25.80±3.16 | 25.52±3.02 | 26.51±3.22 | 26.12±3.26 | <0.001 |
| Hypertension, % | 691 (62.59) | 229 (65.43) | 369 (70.55) | 142 (69.61) | 1686 (61.18) | 963 (63.15) | 1439 (67.37) | 919 (65.55) | <0.001 |
| Dyslipidemia, % | 725 (65.67) | 241 (68.86) | 363 (69.41) | 126 (61.76) | 1844 (66.91) | 1031 (67.61) | 1465 (68.59) | 916 (65.34) | 0.204 |
| Diabetes Mellitus, % | 345 (31.25) | 120 (34.29) | 194 (37.09) | 79 (38.73) | 772 (28.01) | 415 (27.21) | 688 (32.21) | 399 (28.46) | <0.001 |
| PAD, % | 29 (2.63) | 13 (3.71) | 13 (2.49) | 12 (5.88) | 78 (2.83) | 40 (2.62) | 57 (2.67) | 33 (2.35) | 0.192 |
| COPD, % | 22 (1.99) | 4 (1.14) | 17 (3.25) | 10 (4.90) | 48 (1.74) | 31 (2.03) | 55 (2.57) | 50 (3.57) | 0.001 |
| Previous MI, % | 265 (24.00) | 90 (25.71) | 103 (19.69) | 51 (25.00) | 535 (19.41) | 312 (20.46) | 342 (16.01) | 229 (16.33) | <0.001 |
| Previous Stroke, % | 116 (10.51) | 39 (11.14) | 65 (12.43) | 37 (18.14) | 249 (9.03) | 150 (9.84) | 227 (10.63) | 176 (12.55) | <0.001 |
| Current/Ever-Smoker, % | 652 (59.06) | 201 (57.43) | 336 (64.24) | 135 (66.18) | 1601 (58.09) | 794 (52.07) | 1316 (61.61) | 817 (58.27) | <0.001 |
| Previous PCI, % | 345 (31.25) | 106 (30.29) | 131 (25.05) | 49 (24.02) | 623 (22.61) | 393 (25.77) | 457 (21.40) | 314 (22.40) | <0.001 |
| Previous CABG, % | 50 (4.53) | 15 (4.29) | 18 (3.44) | 13 (6.37) | 88 (3.19) | 83 (5.44) | 78 (3.65) | 62 (4.42) | 0.011 |
| Clinical Presentation, % |  |  |  |  |  |  |  |  |  |
| Acute Coronary Syndrome | 716 (64.86) | 212 (60.57) | 405 (77.44) | 154 (75.49) | 1763 (63.97) | 950 (62.30) | 1571 (73.55) | 1025 (73.11) | <0.001 |
| Stable Angina Pectoris | 388 (35.14) | 138 (39.43) | 118 (22.56) | 50 (24.51) | 993 (36.03) | 575 (37.70) | 565 (26.45) | 377 (26.89) |  |
| LVEF, % | 63.76±6.63 | 63.31±6.95 | 61.82±7.69 | 61.55±7.93 | 63.55±6.82 | 63.24±7.12 | 62.07±7.46 | 61.79±7.86 | <0.001 |
| Lp(a), mg/dL | 8.44 [4.15, 15.03] | 49.81 [37.93, 67.86] | 9.38 [5.00, 16.73] | 51.32 [40.58, 68.28] | 10.07 [5.04, 17.45] | 55.52 [40.72, 80.97] | 12.09 [6.47, 19.44] | 55.27 [39.51, 81.43] | <0.001 |
| hs-CRP, mg/L | 0.76 [0.44, 1.19] | 0.75 [0.41, 1.23] | 4.30 [2.73, 9.84] | 4.22 [2.92, 8.76] | 0.92 [0.56, 1.37] | 0.98 [0.57, 1.41] | 4.27 [2.79, 9.50] | 4.92 [2.98, 10.66] | <0.001 |
| LDL-C, mmol/L | 1.45±0.25 | 1.53±0.21 | 1.47±0.26 | 1.52±0.21 | 2.69±0.79 | 2.78±0.80 | 2.81±0.77 | 2.98±0.91 | <0.001 |
| HDL-C, mmol/L | 0.99±0.28 | 1.03±0.31 | 0.90±0.27 | 0.94±0.25 | 1.07±0.28 | 1.12±0.29 | 0.99±0.26 | 1.01±0.26 | <0.001 |
| TG, mmol/L | 1.59±1.23 | 1.34±0.78 | 1.97±1.79 | 1.43±0.78 | 1.85±1.09 | 1.65±0.74 | 1.98±1.13 | 1.77±0.82 | <0.001 |
| TC, mmol/L | 3.02±0.49 | 3.08±0.42 | 3.13±0.67 | 3.05±0.39 | 4.41±0.96 | 4.51±0.93 | 4.56±0.95 | 4.70±1.05 | <0.001 |
| Apo A1, g/L | 1.31±0.24 | 1.31±0.24 | 1.24±0.23 | 1.22±0.22 | 1.39±0.25 | 1.41±0.26 | 1.33±0.24 | 1.32±0.25 | <0.001 |
| Apo B, g/L | 0.57±0.11 | 0.62±0.11 | 0.61±0.12 | 0.62±0.11 | 0.86±0.22 | 0.92±0.23 | 0.90±0.22 | 0.98±0.26 | <0.001 |
| Glucose, mmol/L | 5.78±1.65 | 5.69±1.61 | 6.35±2.29 | 5.98±1.88 | 6.05±1.91 | 5.97±1.85 | 6.57±2.36 | 6.41±2.37 | <0.001 |
| eGFR, ml/min | 92.57±14.01 | 92.91±13.84 | 89.36±16.05 | 87.03±17.08 | 92.87±13.73 | 91.80±13.75 | 90.51±16.27 | 89.78±16.60 | <0.001 |
| Lesion Vessels | 1.33±0.60 | 1.38±0.64 | 1.41±0.73 | 1.38±0.71 | 1.39±0.65 | 1.41±0.64 | 1.43±0.68 | 1.46±0.70 | <0.001 |
| SYNTAX Score^a^ | 10.65±7.71 | 12.00±7.81 | 11.71±8.42 | 12.32±8.40 | 11.31±7.77 | 11.59±8.17 | 11.99±8.22 | 12.79±8.60 | <0.001 |
| Number of Stents | 1.69±1.06 | 1.77±1.06 | 1.88±1.22 | 1.80±1.25 | 1.80±1.11 | 1.80±1.05 | 1.81±1.10 | 1.88±1.15 | 0.004 |
| Medication at Discharge, % |  |  |  |  |  |  |  |  |  |
| Aspirin | 1091 (98.82) | 344 (98.29) | 519 (99.24) | 199 (97.55) | 2732 (99.13) | 1511 (99.08) | 2110 (98.78) | 1389 (99.07) | 0.350 |
| Clopidogrel | 1101 (99.73) | 350 (100.00) | 522 (99.81) | 202 (99.02) | 2753 (99.89) | 1519 (99.61) | 2134 (99.91) | 1399 (99.79) | 0.087 |
| Calcium Channel Blocker | 521 (47.19) | 184 (52.57) | 255 (48.76) | 93 (45.59) | 1356 (49.20) | 750 (49.18) | 1052 (49.25) | 683 (48.72) | 0.740 |
| Beta-Blocker | 973 (88.13) | 313 (89.43) | 485 (92.73) | 189 (92.65) | 2458 (89.19) | 1405 (92.13) | 1945 (91.06) | 1279 (91.23) | 0.001 |
| Statin | 1104 (100.00) | 350 (100.00) | 523 (100.00) | 204 (100.00) | 2756 (100.00) | 1525 (100.00) | 2136 (100.00) | 1402 (100.00) | 1.000 |

Meanings of the abbreviations are identical to those in Table S1.

^a^Calculated using an online calculator (http://www.syntaxscore.com) by a dedicated research group blinded to the clinical data.

**Table S5.** Univariable Cox regression for primary outcome

| **Variables** | **HR** | **95% CI** | **P value** |
| --- | --- | --- | --- |
| Age, years | 1.074 | 1.063‒1.086 | <0.001 |
| Sex, % | 0.843 | 0.665‒1.068 | 0.157 |
| BMI, kg/m^2^ | 0.958 | 0.927‒0.990 | 0.012 |
| Hypertension, % | 1.541 | 1.218‒1.948 | <0.001 |
| Dyslipidemia, % | 0.908 | 0.731‒1.128 | 0.384 |
| Diabetes Mellitus, % | 1.333 | 1.075‒1.654 | 0.009 |
| PAD, % | 1.695 | 1.027‒2.798 | 0.039 |
| COPD, % | 3.066 | 2.026‒4.640 | <0.001 |
| Previous MI, % | 1.430 | 1.127‒1.814 | 0.003 |
| Previous Stroke, % | 1.421 | 1.057‒1.911 | 0.020 |
| Current/Ever-Smoker, % | 1.029 | 0.834‒1.271 | 0.789 |
| Previous PCI, % | 1.416 | 1.132‒1.772 | 0.002 |
| Previous CABG, % | 1.966 | 1.328‒2.910 | 0.001 |
| Clinical Presentation (ACS vs SAP) | 1.153 | 0.919‒1.448 | 0.219 |
| LVEF, % | 0.960 | 0.948‒0.972 | <0.001 |
| Lp(a), mg/dL | 1.004 | 1.000‒1.007 | 0.035 |
| hs-CRP, mg/L | 1.057 | 1.033‒1.081 | <0.001 |
| LDL-C, mmol/L | 0.900 | 0.799‒1.014 | 0.083 |
| HDL-C, mmol/L | 1.609 | 1.135‒2.281 | 0.008 |
| TG, mmol/L | 0.881 | 0.783‒0.991 | 0.034 |
| TC, mmol/L | 0.944 | 0.855‒1.041 | 0.249 |
| Apo A1, g/L | 1.223 | 0.813‒1.838 | 0.334 |
| Apo B, g/L | 0.634 | 0.408‒0.986 | 0.043 |
| Glucose, mmol/L | 1.089 | 1.047‒1.133 | <0.001 |
| eGFR, ml/min | 0.969 | 0.963‒0.974 | <0.001 |
| Lesion Vessels | 0.851 | 0.717‒1.010 | 0.066 |
| SYNTAX Score | 1.004 | 0.992‒1.017 | 0.511 |
| Number of Stents | 0.948 | 0.860‒1.044 | 0.278 |

CI, confidence interval; HR, hazard ratio; meanings of other abbreviations are identical to those in Table S1.

**Table S6.** Association of MACE with Lp(a) grouped by LDL-C or hs-CRP levels alone

| LDL-C Categories | Lp(a) Categories | Events/Total (%) | Multivariable-Adjusted HR (95% Cl) |
| --- | --- | --- | --- |
| Overall |  |  |  |
|  | Lp(a) < 30 mg/dL | 717/6519 (11.0) | 1.000 (reference) |
|  | Lp(a) ≥ 30 mg/dL | 428/3481 (12.3) | 1.104 (0.976‒1.250) |
| LDL-C < 70 mg/dL |  |  |  |
|  | Lp(a) < 30 mg/dL | 170/1627 (10.4) | 1.000 (reference) |
|  | Lp(a) ≥ 30 mg/dL | 61/554 (11.0) | 0.969 (0.716‒1.311) |
| LDL-C ≥ 70 mg/dL |  |  |  |
|  | Lp(a) < 30 mg/dL | 547/4892 (11.2) | 1.000 (reference) |
|  | Lp(a) ≥ 30 mg/dL | 367/2927 (12.5) | 1.122 (0.979‒1.287) |
| Hs-CRP < 2 mg/L |  |  |  |
|  | Lp(a) < 30 mg/dL | 409/3860 (10.6) | 1.000 (reference) |
|  | Lp(a) ≥ 30 mg/dL | 217/1875 (11.6) | 1.066 (0.899‒1.263) |
| Hs-CRP ≥ 2 mg/L |  |  |  |
|  | Lp(a) < 30 mg/dL | 308/2659 (11.6) | 1.000 (reference) |
|  | Lp(a) ≥ 30 mg/dL | 211/1606 (13.1) | 1.154 (0.961‒1.384) |

MACE, major adverse cardiovascular events; LDL-C, low-density lipoprotein cholesterol; hs-CRP, high-sensitivity C-reactive protein; Lp(a), lipoprotein (a); HR, hazard ratio; CI, confidence interval.

**Table S7.** Association of MACE with Lp(a) grouped by concurrent LDL-C and hs-CRP levels

| LDL-C Categories | hs-CRP Categories | Lp(a) Categories | Events/Total (%) | Multivariable-Adjusted HR (95% Cl) |
| --- | --- | --- | --- | --- |
| LDL-C < 70 mg/dL |  |  |  |  |
|  | hs-CRP < 2 mg/L |  |  |  |
|  |  | Lp(a) < 30 mg/dL | 111/1104 (10.1) | 1.000 (reference) |
|  |  | Lp(a) ≥ 30 mg/dL | 30/350 (8.6) | 0.764 (0.503‒1.163) |
|  | hs-CRP ≥ 2 mg/L |  |  |  |
|  |  | Lp(a) < 30 mg/dL | 59/523 (11.3) | 1.000 (reference) |
|  |  | Lp(a) ≥ 30 mg/dL | 31/204 (15.2) | 1.193 (0.751‒1.894) |
| LDL-C ≥ 70 mg/dL |  |  |  |  |
|  | hs-CRP < 2 mg/L |  |  |  |
|  |  | Lp(a) < 30 mg/dL | 298/2755 (10.8) | 1.000 (reference) |
|  |  | Lp(a) ≥ 30 mg/dL | 187/1525 (12.3) | 1.126 (0.933‒1.360) |
|  | hs-CRP ≥ 2 mg/L |  |  |  |
|  |  | Lp(a) < 30 mg/dL | 249/2136 (11.7) | 1.000 (reference) |
|  |  | Lp(a) ≥ 30 mg/dL | 180/1402 (12.8) | 1.120 (0.918‒1.367) |

MACE, major adverse cardiovascular events; LDL-C, low-density lipoprotein cholesterol; hs-CRP, high-sensitivity C-reactive protein; Lp(a), lipoprotein (a); HR, hazard ratio; CI, confidence interval.

**Figure S1.** Prevalence of Lp(a) < 30 mg/dL and Lp(a) ≥ 30 mg/dL by concurrent LDL-C and hs-CRP categories


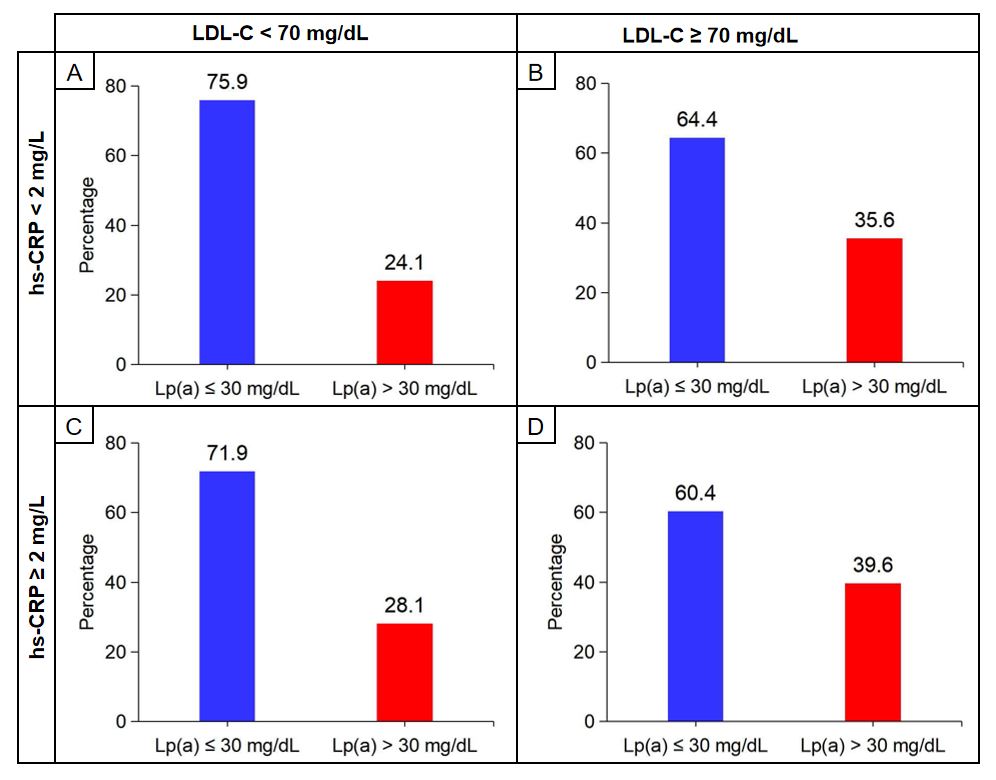


LDL-C, low-density lipoprotein cholesterol; hs-CRP, high-sensitivity C-reactive protein; Lp(a), lipoprotein (a).

**Figure S2.** Cumulative incidence rate of all-cause death with elevated Lp(a) levels according to combinations (LDL-C and hs-CRP categories) during the 5-year follow up.


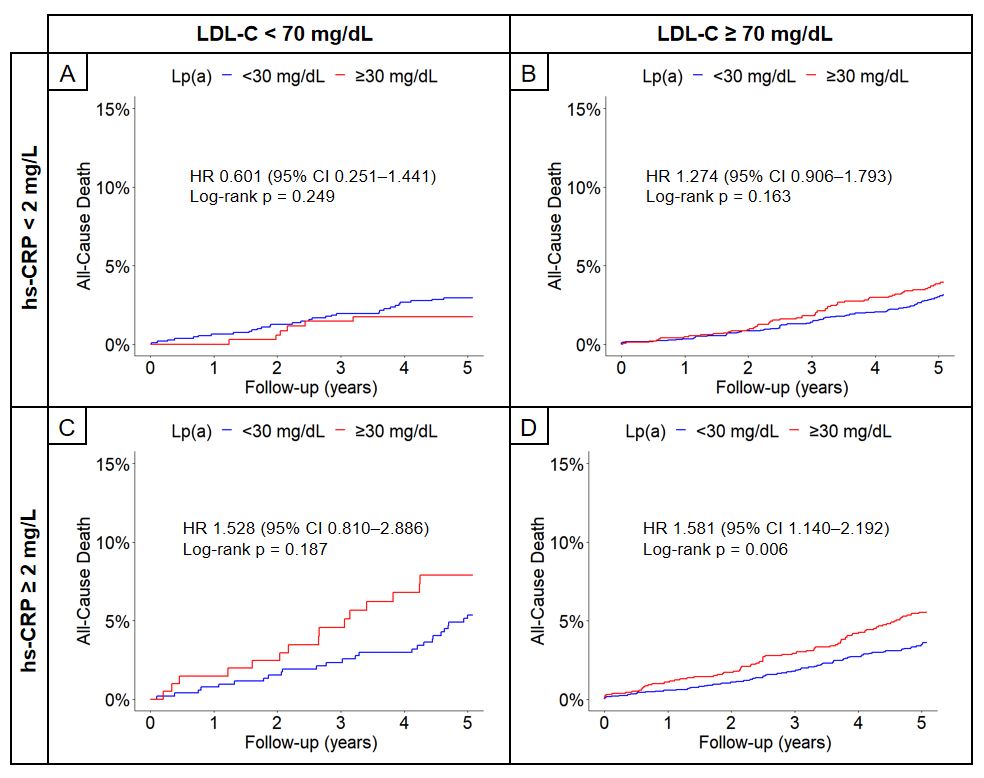


LDL-C, low-density lipoprotein cholesterol; hs-CRP, high-sensitivity C-reactive protein; Lp(a), lipoprotein (a); HR, hazard ratio; CI, confidence interval.
